# Global Variations in Surgical Techniques and Postoperative Care for Radial Forearm Free Flap (RFFF) in Head & Neck Surgery: A Cross-Sectional International Survey

**DOI:** 10.3390/jcm14228023

**Published:** 2025-11-12

**Authors:** Elena Russo, Andrea Costantino, Giannicola Iannella, Filippo Marchi, Antonio Greco, Luca Calabrese, Antonella Polimeni, Remo Accorona, Armando De Virgilio, RFFFSurv Collaborative

**Affiliations:** 1 Department of ‘Organi di Senso’, University “Sapienza”, Viale dell’Università, 33, 00185 Rome, Italy; giannicola.iannella@uniroma1.it (G.I.); antonio.greco@uniroma1.it (A.G.); armando.devirgilio@uniroma1.it (A.D.V.); 2 Otorhinolaryngology Unit, IRCCS Humanitas Research Hospital, Via Manzoni 56, 20089 Rozzano, Milan, Italy; andrea.costantino94@gmail.com; 3 IRCCS Ospedale Policlinico San Martino, 16132 Genoa, Italy; filippo.marchi@unige.it; 4 Department of Otolaryngology-Head and Neck Surgery, Hospital of Bolzano (SABES-ASDAA), Teaching Hospital of the Paracelsus Medical Private University (PMU), 39100 Bolzano, Italy; luca.calabrese@sabes.it; 5 Department of Odontostomatological and Maxillofacial Sciences, Sapienza University of Rome, 00185 Rome, Italy; rettricesapienza@uniroma1.it; 6 Department of Otolaryngology-Head and Neck Surgery, Niguarda Hospital, 20162 Milan, Italy; dott.remoaccorona@gmail.com

**Keywords:** free tissue flap, forearm, radial artery, head, neck, reconstructive surgical procedures, microsurgery, treatment outcome

## Abstract

**Objective**: This cross-sectional survey aimed to comprehensively gather data on radial forearm free flap (RFFF) utilization and practices in head and neck reconstructive surgery. **Methods**: An online questionnaire was organized into seven sections: demographics, surgeon experience, harvesting techniques, microsurgical considerations, postoperative care, flap monitoring, and outcomes. It was distributed by email to 216 head and neck reconstructive surgeons who attended the International Federation of Head and Neck Oncologic Societies (IFHNOS) congress in Rome (21–25 June 2023) using the congress mailing list. Responses were collected from 54 surgeons (25% response rate), representing 15 countries across Europe, Asia, the Americas, and Oceania, underscoring the international scope of the survey between 5 February and 25 March 2024. The questionnaire was not formally piloted or validated. Missing data were managed on a per-question basis. Descriptive statistics were used, and 95% confidence intervals (CIs) were calculated for key surgical outcomes to indicate estimate precision. Associations between categorical variables were analyzed using Pearson’s χ^2^ test with Cramér’s V as an effect size, and relationships between continuous variables were examined using Spearman’s rank correlation (ρ) with 95% confidence intervals (CIs). Given the exploratory design and limited sample size, no correction for multiple comparisons was applied, and the risk of both Type I and Type II errors was acknowledged. **Results**: Variations were observed in harvesting techniques, microsurgical preferences, and postoperative care protocols. Most surgeons initiated flap harvesting concurrently with tumor resection, primarily preserving superficial sensory nerves. Regarding venous outflow, 50% of respondents preferred the cephalic vein, 19% used comitant veins, and 29% utilized both systems when possible. Perioperative antibiotic use was standard practice, though anticoagulant preferences and flap monitoring methods varied. The study achieved a high success rate for RFFF procedures, exceeding 95%, with venous thrombosis identified as the main cause of flap failure. No significant correlations were found between flap failure rate and training method (*p* = 0.21), specialty (*p* = 0.37), annual number of RFFF procedures (*p* = 0.89), surgeon age (*p* = 0.42), or hospital type (*p* = 0.48). Effect sizes were small to moderate, indicating weak or negligible associations. Similarly, perioperative factors such as anticoagulant use (*p* = 0.84), preoperative antibiotics (*p* = 0.42), surgical instruments (*p* = 0.61), suture techniques (*p* = 0.51), and donor vein selection (*p* = 0.20) showed no statistically significant associations with flap loss. Patient satisfaction assessments were inconsistent, with only 39% of surgeons routinely performing them. **Conclusions**: The study provides valuable insights into current RFFF practices and outcomes across an international cohort of head and neck surgeons, highlighting patterns and variability in techniques, perioperative care, and monitoring strategies.

## 1. Introduction

The radial forearm free flap (RFFF) was first described by Yang et al. in 1981 [1], and since then it has proven to be a mainstay flap in head and neck reconstructive surgery. This is attributed to its versatility, thin and pliable soft tissue‚ relative ease of elevation, and consistent vascular anatomy. The extraordinary reliability of the flap is underscored by the presence of a large-caliber artery with numerous perforating vessels that supply a large cutaneous area. Furthermore, the cephalic and comitant veins offer versatile options for venous outflow, individually or in combination, further enhancing the flap’s efficacy. However, this very versatility and vascular anatomy give rise to considerable variations in harvesting and microsurgical techniques among surgeons [2,3,4]. From the initial incision to vessel selection and anastomosis, practices diverge significantly, reflecting the individualized approach required for each patient’s unique anatomy and pathology. Consensus remains elusive regarding the administration of antibiotics, anticoagulants, and flap monitoring modalities [5]. Although RFFF typically achieves success through various postoperative strategies, consistent and effective management of critical factors such as flap monitoring, anticoagulation, and wound care is essential for optimizing patient outcomes. Additionally, donor site morbidity remains a significant concern with traditional RFFF [6,7,8], as patients may experience delayed healing, tendon exposure, functional impairment, or aesthetic issues at the donor site. To address these challenges, techniques such as skin grafting, tissue rearrangement, and innovative wound care methods have been adopted, varying from center to center and further diversifying surgical practices [9,10,11,12,13,14,15,16].

Given the lack of standardization, this study employs a multi-institutional survey of head and neck reconstructive surgeons to characterize current patterns of RFFF use, compare institutional and specialty-specific approaches, and identify perioperative factors associated with surgical success. By defining these trends, the study seeks to guide best-practice development and improved patient outcomes.

## 2. Materials and Methods

This cross-sectional survey employed an online questionnaire created using Microsoft Forms, organized into seven sections. Section I gathered demographic information, such as age, gender, medical specialty, and type of medical center. Section II assessed surgeons’ expertise with RFFF, including years of experience and annual procedure volume. Section III focused on harvesting techniques, including flap harvest timing, incision sites, nerve preservation, and closure techniques. Section IV discussed microsurgical considerations, including artery and vein selection and suture preferences. Section V outlined postoperative care, detailing antibiotic and anticoagulant administration. Section VI examined flap monitoring, including who monitors, frequency, and assessment methods. Finally, Section VII evaluated surgical outcomes and related practices. The full survey questionnaire is available in the Supplementary Material (File S1). Outcome measures included success rates (defined as complete flap survival without major complications), revision rates (any unplanned return to the operating room for flap-related issues), and flap loss rates (total flap failure requiring alternative reconstruction). These outcome measures were self-reported by participants based on their personal clinical experience, without verification through institutional or operative databases. The questionnaire did not specify a defined reporting timeframe (e.g., lifetime vs. recent practice), and responses may therefore reflect aggregated career experience. The section also featured questions about typical postoperative recovery times and factors influencing the need for revisions, based on surgeons’ experiences. Respondents were also asked whether they regularly assess patient satisfaction and, if so, what methods they use. The questionnaire was designed collaboratively by two experienced head and neck reconstructive surgeons to ensure content relevance and clarity. Free-text responses were reviewed independently by two authors and categorized thematically by consensus without formal qualitative coding, given the exploratory nature of the study. The questionnaire was not formally piloted or validated before distribution and missing or non-analyzable data were managed on a per-question basis, allowing for partial completion while maintaining data integrity. Consequently, denominators may vary between analyses, reflecting the number of valid responses available for each specific question. The survey was conducted among attendees of the recent International Federation of Head and Neck Oncologic Societies (IFHNOS) congress in Rome (21–25 June 2023), which was attended by surgeons from over 50 countries. Using the congress mailing list, the questionnaire was emailed to 216 head and neck reconstructive surgeons registered for the event, ensuring that only experienced clinicians actively involved in practice received the invitation. This approach represents a convenience sample, as participation was limited to congress attendees who voluntarily responded. The survey was conducted from 5 February to 25 March 2024. Responses were requested in multiple formats, such as multiple-choice, yes/no, and free text, providing flexibility for participants.

The data were summarized using descriptive statistics. Dichotomous variables were presented as counts and percentages, while continuous variables were expressed as mean and standard deviation (SD), or as median and interquartile range (IQR) if the values were not normally distributed at the Shapiro–Wilk normality test. For the primary surgical outcomes (success rate, revision rate, and flap loss rate), 95% confidence intervals (CIs) were calculated to reflect the precision of these estimates. Stratified analyses of these outcomes by hospital type or surgeon experience were not performed due to small subgroup sizes, which would compromise statistical reliability. Associations between categorical variables, including perioperative practices (anticoagulant use, antibiotic prophylaxis, suture type, donor vein selection) and surgeon- or institution-related characteristics (training method, specialty, hospital type), were analyzed using Pearson’s χ^2^ test with Cramér’s V as a measure of effect size. When expected cell counts were below five, categories were collapsed to meet χ^2^ assumptions, or results were interpreted descriptively.

Relationships between continuous variables (e.g., failure rate, surgeon age, annual number of RFFF procedures) were examined using Spearman’s rank correlation coefficient (ρ) with 95% confidence intervals (CIs) derived via Fisher’s z-transformation. Given the exploratory nature of the study and the limited sample size, no correction for multiple comparisons was applied, and the risk of both Type I and Type II errors was acknowledged. All analyses were carried out using STATA version 18 software (2023 StataCorp LLC. 4905 Lakeway Drive, College Station, TX, USA), with statistical significance defined as *p* < 0.05.

## 3. Results

### 3.1. Participants

**Figure 1:**
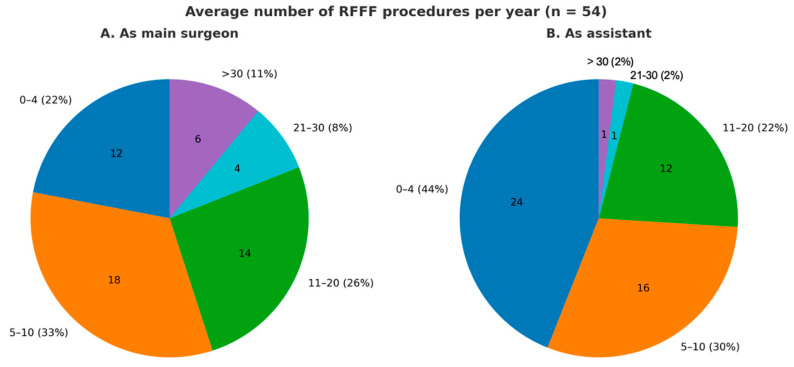
Pie charts illustrating the average annual number of procedures performed by each participant, categorized by role: as the main surgeon and as an assistant.

The demographic data of the survey participants are presented in Table 1. Out of 216 head and neck surgeons invited, 54 responded to the survey, giving a 25% response rate. Of these respondents, 48 (89%) were male. Among them, 39 (64%) were ENT surgeons, 11 (18%) maxillofacial surgeons, 2 (3%) plastic surgeons, and 9 (15%) from other departments. The average age was 46 years (±9.97). Most were academic (69%; *n* = 37) and fellowship-trained (63%; *n* = 34). Survey respondents represented 15 countries, including Italy, Australia, Brazil, Canada, China, Colombia, Germany, India, Japan, the Netherlands, Spain, Switzerland, Thailand, the United Kingdom, and the United States, indicating broad international distribution. One-third practiced in Italy. Additionally, more than half (59%; *n* = 32) had over 10 years of experience in head and neck reconstructive surgery. The average number of procedures per year for each participant is shown in Figure 1.

### 3.2. Harvesting Technique

Most surgeons (83%; *n* = 45) initiate flap harvesting simultaneously with tumor resection. Additionally, 70% of respondents (*n* = 38) start the skin incision on the radial side. Most respondents (87%; *n* = 47) preserve superficial sensory nerves to reduce donor-site morbidity. The main reasons for not preserving the nerves include their proximity to the cephalic vein and radial artery perforators, as well as the need for a larger skin paddle. Only one surgeon mentioned that the decision to preserve the nerves depends on the time available for dissection. About one-third of respondents (*n* = 18) repair damaged sensory nerves during the harvesting process.

**Figure 2:**
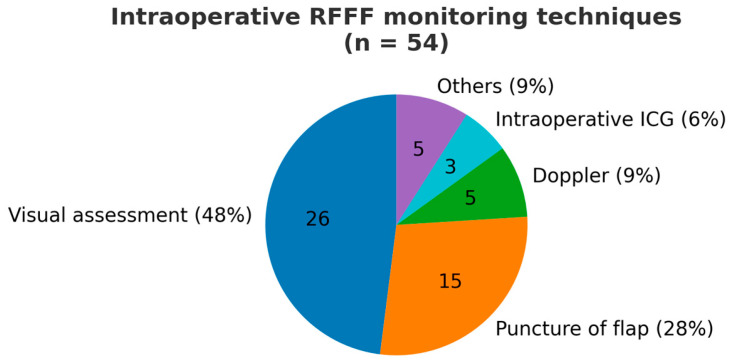
Pie chart showing the different modalities utilized for monitoring the flap’s viability during the harvesting procedure.

Regarding vein preparation during RFFF harvesting, 83% of surgeons (*n* = 45) consistently prepare the cephalic vein, but only about half (*n* = 27) preserve the fat between the cephalic vein and the pedicle. Those who preserve the fat argue it protects the pedicle during dissection, prevents kinking after insetting, and adds bulk for reconstruction. In contrast, others believe removing the fat allows for better vessel positioning without threatening flap viability. Figure 2 illustrates the modalities used to monitor the flap’s viability during the harvesting procedure. Most surgeons monitor flap viability through visual assessment and puncture, with only 9% using intraoperative Doppler and 7% using indocyanine green (ICG) angiography. Forty percent (*n* = 22) determine the length of the pedicle based on the recipient site anatomy post-resection.

**Figure 3:**
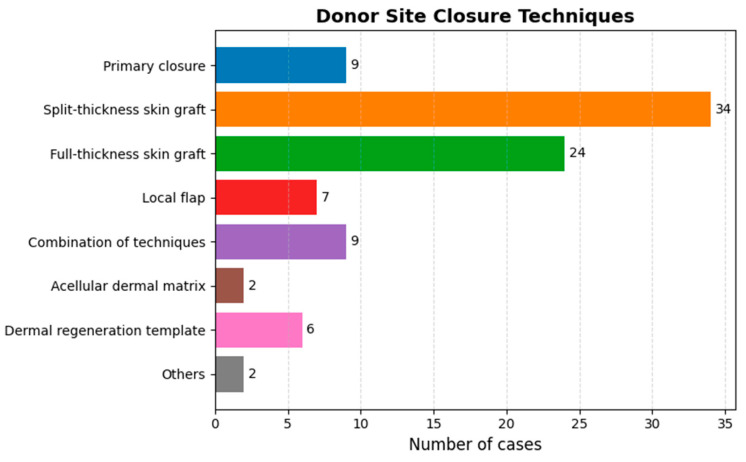
Bar chart presenting the various techniques employed to close the donor site following flap harvesting.

Figure 3 illustrates the various techniques used to close the donor site after flap harvesting. The primary method is split-thickness skin grafting, selected by 34 respondents, followed by full-thickness skin grafting, chosen by 24. Additionally, 9 surgeons advocate for primary closure in selected cases. Respondents indicated that achieving a tension-free primary closure depends on patient- and flap-related factors such as forearm size, skin laxity and thickness, the amount of subcutaneous fat, limited flap size, and the use of either a fascial (rather than fasciocutaneous) flap or an alternative flap design. Seventy-eight percent (*n* = 42) use static pressure dressings for the donor site, while 22% (*n* = 12) prefer negative pressure dressings.

### 3.3. Microsurgery

**Figure 4:**
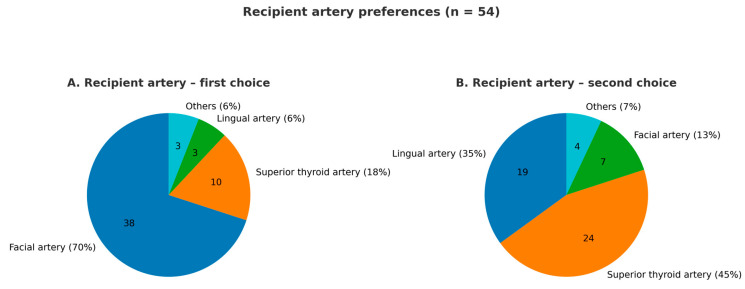
Pie charts displaying the preferences of survey respondents regarding recipient arteries.

Figure 4 summarizes the preferences of survey respondents for recipient arteries. The facial artery was the most commonly selected (70%; *n* = 38), followed by the superior thyroid artery (44%; *n* = 24). Notably, 46% (*n* = 25) of respondents opened the arterial anastomosis immediately after completion, with 64% of these allowing venous discharge through a second vein during the venous anastomosis. The cephalic vein was the preferred donor vein for 50% (*n* = 27) of respondents, while 19% (*n* = 10) chose the comitant veins. Additionally, 29% (*n* = 16) reported routinely using both venous systems whenever feasible, considering factors such as comitant vein caliber, suitability for matching recipient veins, and the presence of two recipient veins in the neck. One surgeon noted always attempting to use the confluence between the cephalic and comitant veins. Overall, 76% (*n* = 41) prioritized vein diameter, most commonly 2 mm or larger, as the main criterion for selection. Additionally, 74% (*n* = 40) preferred end-to-end anastomosis, largely due to its ease, while 18% (*n* = 10) chose end-to-side. Most surgeons used 8 or 9–0 nylon or prolene sutures, with 78% (*n* = 42) favoring interrupted sutures. Regarding visualization tools, 88% (*n* = 48) preferred using a microscope, while 8% (*n* = 4) opted for alternatives like the exoscope or loupes.

### 3.4. Postoperative Care and Flap Monitoring Modalities

**Figure 5:**
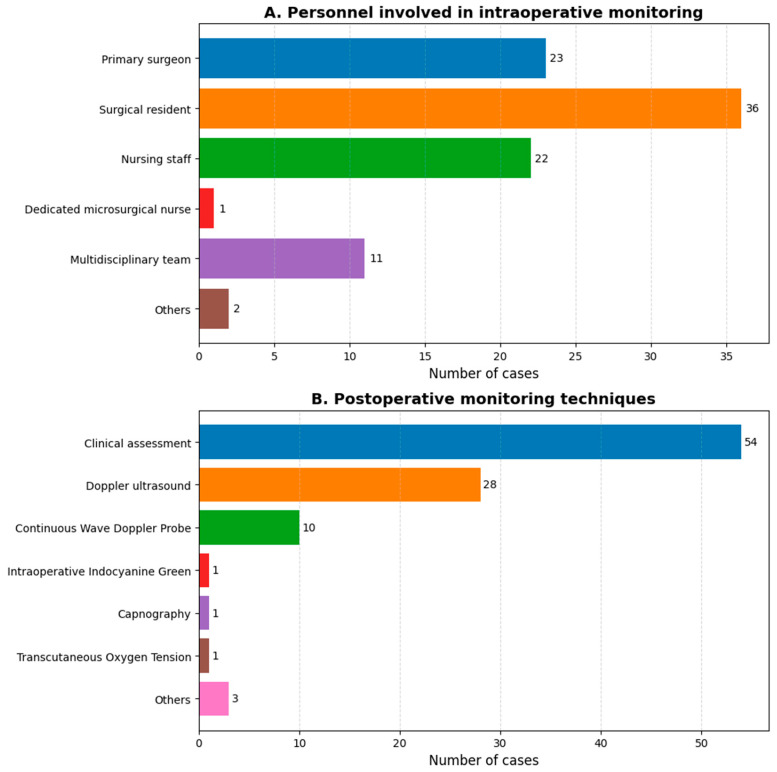
Bar chart showing (**A**) the individuals responsible for monitoring the flap post-surgery, and (**B**) the strategies employed for this monitoring.

Of the survey respondents, 94% (*n* = 51) routinely administer perioperative antibiotics. Specifically, 16 surgeons (33%) use antibiotics for less than 24 h, 9 surgeons (18%) for 48 h, 8 surgeons (16%) for 2 days to 1 week, and 16 surgeons (33%) for at least 1 week. Regarding anticoagulant use, 59% of respondents administer them, with 66% (*n* = 23) using heparin; only 30% use it at a therapeutic dose. Additionally, 6 surgeons (17%) use acetylsalicylic acid or both heparin and acetylsalicylic acid, sometimes simultaneously. Almost half (47%; 17 out of 35) start anticoagulants immediately after surgery, while 39% begin on postoperative day 1. Duration of therapy varies, with 38% indicating less than 1 week, 31% between 1 and 2 weeks, and 31% more than 2 weeks. Figure 5 illustrates who is responsible for monitoring the flap after surgery, along with the employed strategies. Monitoring strategies for buried flaps include options like an implantable Doppler or a small skin paddle. Most respondents monitor the flap closely within the first 24–48 h post-surgery, with checks every hour to every 4 h.

### 3.5. Outcomes

Among 40 respondents, 68% (95% CI: 52–81%) reported a success rate >95%, with an overall revision rate of 5% (95% CI: 2–9%). Only 17% (95% CI: 9–29%) indicated a failure rate above 5%, primarily due to venous thrombosis (80%) and arterial occlusion (20%). Other contributing factors included patients’ comorbidities, neck infections, hematomas or edema, and delays in revision surgery.

To explore potential predictors of flap failure, both categorical and continuous variables were analyzed using Cramér’s V and Spearman’s rank correlation (ρ) with 95% confidence intervals (CIs). No significant correlations were found between failure rate and training method (Cramér’s V = 0.36), specialty (Cramér’s V = 0.25), annual number of RFFF procedures (ρ = −0.02; 95% CI: −0.29 to 0.25), surgeon age (ρ = 0.11; 95% CI: −0.16 to 0.38), or hospital type (university vs. non-university). Effect sizes were small to moderate, indicating weak or negligible associations.

Similarly, no significant relationships were observed between failure rate and perioperative factors, including anticoagulant use (χ^2^ = 0.04, *p* = 0.84, Cramér’s V = 0.05), preoperative antibiotics (χ^2^ = 0.65, *p* = 0.42, Cramér’s V = 0.13), surgical instruments (χ^2^ = 0.98, *p* = 0.61, Cramér’s V = 0.16), suture techniques (χ^2^ = 0.43, *p* = 0.51, Cramér’s V = 0.10), and donor vein selection (χ^2^ = 3.18, *p* = 0.20, Cramér’s V = 0.28).

Confidence intervals were not estimated for these categorical associations due to small subgroup counts, which limit precision. All effect sizes indicated weak or clinically negligible relationships.

Regarding patient satisfaction after RFFF procedures, methods varied: 39% (*n* = 21) conducted routine assessments, 37% (*n* = 20) did not assess, and 24% (*n* = 13) assessed satisfaction in a non-systematic way, using tools like the Manchester Scar Scale and POSAS, as well as verbal discussions.

## 4. Discussion

The RFFF is a key technique in head and neck reconstructive surgery due to its versatility, ease of harvesting, and reliable vascular anatomy. However, there are notable variations in surgical techniques and postoperative care among surgeons.

Most surgeons prefer to harvest the flap simultaneously with tumor resection, starting the incision on the radial side due to the more predictable vascular anatomy. This approach allows quicker access to the radial artery and veins, enhancing safety, but it may increase the risk of damaging the radial nerve and causing sensory deficits. Preserving superficial sensory nerves during harvesting is widely accepted to minimize donor-site morbidity, as reported by 87% of respondents [17]. When the superficial branches of the radial nerve are injured, patients often experience temporary paresthesia and/or pain after surgery. Unless the nerve is entirely resected, these symptoms rarely disrupt daily activities, but some patients may suffer from chronic neuropathic pain [18]. Additionally, young and active patients with paresthesia are at an increased risk of sustaining secondary injuries [19]. Our survey found that the decision not to preserve these nerves is usually influenced by their anatomical relationship to the cephalic vein and radial artery perforators, or the need for an extensive skin paddle. One participant noted that the decision to preserve the nerves depends on the available time for dissection. Nevertheless, considering the literature-reported benefits of reduced morbidity, we believe that preserving the radial sensory nerve during RFF harvest is advisable whenever possible.

The survey reveals inconsistency in handling the cephalic vein and surrounding fat during RFFF harvesting, with only half of the surgeons consistently preserving it. While some focus on protecting the pedicle and adding bulk, others opt for more flexibility in vessel positioning. These findings suggest a range of techniques are in use, each with potential implications for flap viability and outcomes. Preserving surrounding fat appears to be a common practice unless it interferes with vessel placement. Ultimately, technique selection often depends on the specific clinical scenario, individual patient anatomy, and the surgeon’s experience.

Regarding microsurgical techniques, the survey reveals a preference for the facial and superior thyroid arteries as recipient arteries. Interestingly, many surgeons prefer to open the arterial anastomosis immediately after completing it. This practice is likely driven by the need to promptly verify the patency of the anastomosis and to ensure adequate arterial inflow before proceeding to the venous anastomosis. However, establishing arterial flow before completing the venous pedicle anastomosis can cause a brief venous stasis [20,21,22]. This stasis is believed to cause more damage to microsurgical flaps than arterial occlusion due to an increase in platelet and fibrinogen accumulation, higher intravascular pressure, and subsequent interstitial edema, which may lead to vasoconstriction and slow venous reflow [23,24]. Despite this concern, it is noteworthy that among those who open the arterial anastomosis right after its completion, 64% indicated they allow venous discharge through a second vein while performing the venous anastomosis, and 20% allow it intermittently, likely to avoid venous stasis. Nevertheless, an animal study by Zhang et al. [25] demonstrated that a brief venous stasis during anastomosis, after establishing arterial inflow, is not detrimental to flap survival.

Concerning vein selection for microvascular anastomosis, half of the respondents prefer using the cephalic vein as their primary donor vein, 19% opt for the comitant veins, while 29% use both the cephalic and comitant veins. The cephalic vein is favored due to its ease of harvest and microanastomosis, being a large, thick-walled vein located consistently beneath the subcutaneous fat [26]. Conversely, anastomosing comitant veins can be challenging due to their small caliber. However, this difficulty can be addressed by harvesting the coalesced vein, which originates from the convergence of comitant veins at the proximal end of the radial artery, near the bifurcation point of the brachial artery. The main advantage of the coalesced vein is its lumen width, which is nearly equivalent to that of the cephalic vein, making it easier to work with. This is particularly important because venous thrombus formation, a common cause of flap failure, often originates from the superficial venous system [27]. Additionally, cephalic vein occlusion from previous intravenous cannulation can lead to flap failure despite appearing normal during harvesting [28]. Thus, the feasibility of relying solely on the superficial vein system is debated. Recent meta-analyses have further examined this controversy. Bai et al. [29] found that performing dual venous anastomoses involving both superficial and deep systems tends to reduce venous compromise, although the difference did not reach statistical significance. Similarly, Xie et al. [30] compared superficial versus deep single-system anastomoses and reported that venous compromise was more common in the superficial system group, though the evidence was insufficient to conclusively favor one system over the other. These findings reinforce the notion that the optimal drainage strategy remains unsettled, likely depending on intraoperative factors such as vessel quality, flow dynamics, and surgeon preference. From this perspective, the need for single versus dual venous anastomosis also arises. Interestingly, about one-third of our survey respondents tend to perform sequential anastomoses on multiple veins when dealing with comitant veins. The decision to perform sequential anastomosis is mainly influenced by vein size and blood flow. While dual anastomosis creates two separate perfusion paths, potentially enhancing safety, it may also reduce perfusion pressure, slow blood flow velocity, and increase the risk of vein thrombosis [31]. Nonetheless, a recent study [32] demonstrated that dual venous anastomosis could reduce venous compromise resulting from unexpected causes in RFFF transfer. Moreover, hemodynamic studies have shown that deep veins have twice the drainage volume per unit time compared to superficial veins, highlighting their superior capacity [33]. The vascular network of a fasciocutaneous flap comprises numerous arterial and venous connections within the fascial layer, known as the septocutaneous vascular network. This network originates from arterial perforators and their accompanying comitant veins.

Postoperative care protocols exhibit significant variability, particularly in administering antibiotics and anticoagulants. While a high percentage of respondents routinely administer perioperative antibiotics, the duration of administration varies widely, reflecting differing interpretations of prophylactic guidelines. The perceived need for prolonged antibiotic intake remains high. However, recent publications have questioned this trend, indicating that long-term antibiotic use may not better prevent surgical site infections [34,35]. Specific antibiotic choices, such as the combination of amoxicillin/ampicillin and sulbactam or cephalosporins, have been suggested to be more critical than the duration of administration for preventing surgical site infections [35]. Similarly, the use of anticoagulants, predominantly heparin, varies in terms of timing and duration, with no significant correlation found between anticoagulant use and flap failure rates. Previous studies have found that heparin use does not significantly influence flap viability or complication rates, and higher doses may even be associated with greater flap loss [36,37,38]. Some experts advocate for the combined use of aspirin and subcutaneous heparin [39], while others suggest that low-dose heparin or aspirin prophylaxis is preferable [40]. The routine administration of anticoagulants remains debated, with some researchers arguing that the risks may outweigh the benefits [41]. From this perspective, there is a need for greater uniformity in these protocols to enhance patient outcomes and reduce the risk of complications.

Flap monitoring practices vary significantly among surgeons. Most rely on visual assessment and flap puncture, while a smaller proportion utilizes intraoperative Doppler and indocyanine green (ICG) angiography. Lately, there has been a shift toward using innovative techniques in this field, such as implantable Doppler systems and thromboelastometry [42]. More advanced methods, like near-infrared spectroscopy and hemoglobin oxygenation measurement, have also been found to be valuable additions to traditional monitoring techniques [42]. These advanced methods are particularly useful in the early detection of microvascular thrombosis. More standardized and reliable monitoring techniques are needed, especially for buried flaps where traditional methods are impractical.

The high success rate reported by most respondents (>95%) aligns with existing literature supporting RFFF reliability. However, the revision and failure rates underscore the persistent challenges associated with venous thrombosis and arterial occlusion, which are the primary causes of flap failure. Nevertheless, our survey did not identify any procedural, surgeon-related, or institutional predictors of flap failure. These findings suggest that outcomes are multifactorial, likely reflecting complex interactions between patient-related factors, perioperative management, and institutional practices rather than single determinants.

Finally, patient satisfaction assessments are often inconsistent, with many not conducting any evaluations. Although some surgeons use structured questionnaires, such as POSAS and EORTC QoL [43,44], to measure patient-reported outcomes, the variability in assessment practices highlights the need for standardized methods to evaluate satisfaction and quality of life after reconstruction. Currently, this heterogeneity limits meaningful comparisons across institutions and complicates the interpretation of patient-reported results within wider quality improvement efforts. Future collaborative initiatives may help clarify how standardized assessment tools can enhance comparability while preserving local clinical flexibility.

In conclusion, this study offers valuable insights into the use of RFFF in head and neck reconstructive surgery, but some limitations need to be acknowledged. As a self-reported survey, the findings rely on the accuracy and sincerity of participants’ responses, which might reflect individual practices rather than official institutional protocols. The key outcome measures (success rate, revision rate, and flap loss rate) were self-reported and not verified against institutional records, and the absence of a defined reference period (e.g., recent practice vs. lifetime experience) may introduce recall bias. The recruitment strategy, based on attendees of a single international congress, represents a convenience sample that, while providing access to a broad professional audience, may have led to professional and institutional clustering. This approach limits the external validity of the findings, as it may not fully reflect global reconstructive practices. Voluntary participation may also introduce selection bias, as surgeons who chose to respond may differ in experience or interest from those who did not, and the survey was not randomized. Another limitation is that the survey questionnaire, although carefully designed by experienced head and neck surgeons, was not formally piloted or validated before distribution. The relatively small number of respondents further limits statistical power, increasing the likelihood of Type II error, particularly in subgroup analyses; therefore, the statistical findings should be interpreted with caution, especially since adjustments for multiple comparisons were not applied, given the exploratory nature of the study and the use of aggregated self-reported data rather than patient-level outcomes. Geographic distribution may also affect generalizability, with about one-third of respondents practicing in Italy. Additionally, only 63% of participants completed fellowship training, but in some countries, equivalent expertise can be attained without a formal fellowship. The survey, designed by head and neck surgeons, may have introduced selection bias and resulted in a low response rate from other departments. Variations in healthcare systems also limit the inclusion of relevant medical units, further contributing to selection bias. Furthermore, the presence of open-ended questions, while enriching qualitative data, complicates analysis. Therefore, while the findings offer useful insights, they should be interpreted cautiously, considering these limitations.

## 5. Conclusions

This study emphasizes the complexity and variability of RFFF procedures for head and neck reconstruction. The different techniques for harvesting, microsurgery, postoperative care, and monitoring reflect the individualized and experience-driven nature of these surgeries. Although most respondents reported high success rates, the variation in perioperative management indicates the necessity for further multicenter, data-driven research. Our findings offer a descriptive overview of current international practices and could provide a foundation for future collaborative efforts to develop consensus-based protocols and improve the comparability of outcomes.

## Figures and Tables

**Table 1:** Survey participants’ demographic data. Abbreviations: SD standard deviation, ENT ear, nose, and throat, H&N head and neck, RFFF radial forearm free flap.

Characteristic	
Gender	
Male	48 (88.8%)
Female	4 (7.5%)
Not reported	2 (3.7%)
Age (mean ± SD)	46 ± 9.97
Country	
Australia	1 (1.8%)
Brazil	4 (7.5%)
Canada	2 (3.7%)
China	1 (1.8%)
Colombia	2 (3.7%)
Germany	4 (7.5%)
India	2 (3.7%)
Italy	19 (35.2%)
Japan	1 (1.8%)
Netherlands	3 (5.5%)
Spain	4 (7.5%)
Switzerland	1 (1.8%)
Thailand	2 (3.7%)
UK	2 (3.7%)
USA	6 (11.1%)
Type of medical center	
University	37 (68.5%)
Non-university	17 (31.5%)
Specialization	
ENT	39 (64%)
Plastic Surgery	2 (3%)
Maxillofacial Surgery	11 (18%)
Other	9 (15%)
Type of training	
Residency training	21 (38.9%)
Fellowship training	34 (62.9%)
Attending dedicated workshops and courses	25 (46.3%)
Simulation training	6 (11.1%)
Mentorship	13 (24.1%)
Other	2 (3.7%)
Experience in H&N reconstructive surgery (years)	
<1	3 (5.5%)
1–2	1 (1.8%)
2–5	7 (13.0%)
5–10	11 (20.4%)
>10	32 (59.3%)
Average number of RFFF as main surgeon	
0–4	12 (22.2%)
5–10	18 (33.3%)
11–20	14 (26.0%)
21–30	4 (7.4%)
>30	6 (11.1%)
Average number of RFFF as assistant	
0–4	24 (44.5%)
5–10	16 (29.6%)
11–20	12 (22.3%)
21–30	1 (1.8%)
>30	1 (1.8%)

## Data Availability

The data presented in this study are available on request from the corresponding author.

## Supplementary Materials

The following supporting information can be downloaded at: https://www.mdpi.com/article/10.3390/jcm14228023/s1, File S1. Final Survey Questionnaire on Radial Forearm Free Flap (RFFF) Practices; File S2. RFFFSurv Collaborative.
