# Supplementary material for: Global Variations in Surgical Techniques and Postoperative Care for Radial Forearm Free Flap (RFFF) in Head & Neck Surgery: A Cross-Sectional International Survey
Source: J Clin Med. 2025 Nov 12;14(22):8023. doi: 10.3390/jcm14228023 (PMC12653973; doi:10.3390/jcm14228023)
Supplement: Supplementary file 1 [file jcm-14-08023-s001.zip › jcm-3945282-supplementary file S2. RFFFSurv Collaborative.pdf]

<u>RFFFSurv Collaborative:</u>

Luiz P Kowalski MD, Head and Neck Surgery, Faculty of Medicine, University of Sao Paulo, Sao Paulo, Brazil; **Davide Di Santo MD**, Otorhinolaryngology Head and Neck Surgery, University Hospitals Leuven, Leuven, Belgium; **Alvaro Sanabria MD**, Department of Surgery, School of Medicine, Universidad de Antioquia/Hospital Universitario San Vicente Fundación-CEXCA Centro de Excelencia en Enfermedades de Cabeza y Cuello, Medellín, Colombia; **Bruce Ashford MD**, University of Wollongong, Wollongong, Australia; **Marco Antonio Mascarella MD**, Department of Otolaryngology - Head and Neck Surgery, McGill University, Montreal, Quebec; **Alex Marcin Mlynarek MD**, Department of Otolaryngology - Head and Neck Surgery, McGill University, Montreal, Quebec; **Xiaohong Chen MD**, Beijing Tongren Hospital, Capital Medical University, No. 1 Dongjiaomin Lane, Dongcheng District, Beijing 100730, China; **Davin Yavapolkul MD**, Rangsit University, Thailand; **Arjun Gurmeet Singh MD**, Tata Memorial Centre and HBNI, Mumbai, India; **Achille Tarsitano MD**, Oral and Maxillo-facial Surgery - Alma Mater Studiorum University of Bologna, IRCCS Azienda Ospedaliera Universitaria di Bologna; **Michiel van den Brekel MD**, Netherlands Cancer Institute - Antoni van Leeuwenhoek Amsterdam, The Netherlands; **Orlando Guntinas-Lichius MD**, Department of Otorhinolaryngology, Institute of Phoniatry/Pedaudiology, Jena University Hospital, Jena, Germany; **Francesco Riva MD**, The Royal Marsden NHS Foundation Trust, London UK; **Luca de Campora MD PhD**, St. John Addolorata Hospital, Rome;.

David Virós Porcuna MD, Otorhinolaryngology Department, Hospital Germans Trias, I Pujol, Universitat Autòonoma de Barcelona, Badalona, Spain; **Andres Chala MD**, University of Caldas, Manizales, Caldas, Colombia, South America; **Pietro Salvatori MD** Head & Neck Surgeon (Ret.), Milan, Italy; **Antonio Marí-Roig MD**, Department of Maxillofacial Surgery, Bellvitge University Hospital, L'Hospitalet de Llobregrat, 08907 Barcelona, Spain; **Aina Brunet MD**, Department of Maxillofacial Surgery, Bellvitge University Hospital, L'Hospitalet de Llobregrat, 08907 Barcelona, Spain; **Alberto Deganello MD**, Otolaryngology Head and Neck Surgery Department, IRCCS National Cancer Institute (INT), 20133 Milan, Italy; **Pietro De Luca MD**, Isola Tiberina - Gemelli Isola Hospital, Rome, Italy; **Rui Fernandes MD**, Division of Head and Neck Surgery, Department of Oral and Maxillofacial Surgery, University of Florida College of Medicine,-Jacksonville 653-1 West 8th, Street, Jacksonville, FL 32209, USA; **Alfio José Tincani MD**, Faculdade de Ciências Médicas, Universidade Estadual de Campinas, Campinas, SP, Brazil; **Mario Ciniglio Appiani MD**, Department Organs of Sense, ENT Section, 'Sapienza' University of Rome, Viale del Policlinico 155, 00100, Rome, Italy; **Matthaeus Stoehr MD**, Head and Neck Surgery, Department of Otolaryngology, University Hospital Leipzig, 04103 Leipzig, Germany; **Giacomo Colletti MD**, Department of Medical and Surgical Sciences for Children & Adults, Cranio-Maxillo-Facial Surgery, University of Modena and Reggio Emilia, Largo del Pozzo 71, 41124 Modena, Italy; **Richard Shaw MD**, Head and Neck Surgery, Aintree University Hospital, Liverpool, UK; Department of Molecular and Clinical Cancer Medicine, University of Liverpool, Liverpool, UK; **Sudhir Nair MD**, Dept. of Head and Neck Surgery, Tata Memorial Centre, Parel, Mumbai 400012 India; **Leone Giordano MD**, Dept. of Otorhinolaryngology-Head and Neck Surgery, IRCCS San Raffaele, Vita-Salute San Raffaele University, Via

Olgettina 60, Milan, 20132, Italy; **Thadeu Rezende Rangel Fernandes MD**, Universidade de São Paulo, Rua da Rectory, 374 – Cidade Universitária, Butantã, Sao Paulo, SP, Brazil; **Pablo Parente-Arias MD**, Servicio de Otorrinolaringología, Hospital Universitario Lucus Augusti, Lugo, España; **Francesco Maria Egro MD**, Department of Plastic Surgery, University of Pittsburgh, Pittsburgh, PA, USA; **Nausica Montalto MD**, Section of Otorhinolaryngology-Head and Neck Surgery, Department of Neurosciences, University of Padova, Azienda Ospedale Università Padua, Padua, Italy; **Luca Gazzini MD**, Otorhinolaryngology-Head and Neck Surgery Department, San Maurizio Hospital, Bolzano, Italy; **Rita De Berardinis MD**, Unit of Otorhinolaryngology, Department of Clinical Sciences and Translation Medicine, Tor Vergata University, Rome, Italy; **Richard Dirven MD PhD**, Department of Head and Neck Oncology and Surgery, Netherlands Cancer Institute, Amsterdam, the Netherlands; **Bruno Albuquerque Sousa MD**, Instituto Nacional do Câncer Brasileiro, Departamento de Cirurgia de Cabeça e Pescoço, Rio de Janeiro, RJ, Brazil; **Roland Giger MD**, Dept. Otorhinolaryngology, Head and Neck Surgery, Inselspital, Bern University Hospital, University of Bern, Bern, Switzerland; **Matthew Spector MD**, Department of Otolaryngology, Head and Neck Surgery, University of Pittsburgh Medical Center, Pittsburgh, PA 15213, USA; **Francesca Plantone MD**, Otolaryngology and Head and Neck Unit, Ospedale Di Venere, Bari, Italy; **C. René Leemans** MD, Department of Otolaryngology-Head and Neck Surgery, Amsterdam University Medical Center, VU University, P.O. Box 7057, 1007MB Amsterdam, The Netherlands; **Ulrich Kisser MD**, Department of Otorhinolaryngology, Head and Neck Surgery, Martin-Luther-University Halle-Wittenberg, Germany; **Christian Stephan Betz MD**, Department of Otorhinolaryngology, Universitätsklinikum Hamburg-Eppendorf, Hamburg, Germany; **Brett A. Miles MD**, DDS MD - Northwell Cancer Institute, New York NY USA; **Marta Tagliabue MD**, Department of Otorhinolaryngology and Head and Neck Surgery, IEO, European Institute of Oncology IRCCS, Milan, Italy.
