# Supplementary material for: Global Variations in Surgical Techniques and Postoperative Care for Radial Forearm Free Flap (RFFF) in Head & Neck Surgery: A Cross-Sectional International Survey
Source: J Clin Med. 2025 Nov 12;14(22):8023. doi: 10.3390/jcm14228023 (PMC12653973; doi:10.3390/jcm14228023)
Supplement: Supplementary file 1 [file jcm-14-08023-s001.zip › jcm-3945282-supplementary file S1. Final Survey Questionnaire on Radial Forearm Free Flap (RFFF) Practices.pdf]

Appendix A – Final Survey Questionnaire on Radial Forearm Free Flap (RFFF) Practices

This appendix reproduces the questionnaire items included in the analysis. Questions are organized by section and include available response options where present in the original instrument.

Section I – Demographic information

(OP) Age (number)

(ON) Country

(M) Specialization
• ENT
• Plastic surgery
• Maxillofacial surgery
• Other (please specify)

(ON) Type of medical center
• University
• Non-university

Section II – Experience with radial forearm free flap (RFFF)

(ON) Experience in head and neck reconstructive surgery (years)
• <1
• 1-2
• 2-5
• 5-10
• >10

(ON) Experience with RFFF (average number of procedures per year as a main surgeon)
• 0-4
• 5-10
• 11-20
• 21-30
• >30

(ON) Experience with RFFF (average number of procedures per year as an assistant)
• 0-4
• 5-10
• 11-20
• 21-30
• >30

(M) How did/do you learn reconstructive surgery?
• Residency training
• Fellowship training
• Attending dedicated workshops and courses
• Simulation training
• Mentorship
• Other (please specify)

(OP) In which clinical scenarios do you find radial forearm free flap to be most suitable?

Section III – Harvesting technique

(ON) On which side do you typically start the incision for the skin paddle during RFFF harvest?
• Radial side
• Ulnar side

(ON) Do you systematically preserve the superficial sensory nerve during RFFF harvest?
• Yes
• No

(OP) What factors influence your decision regarding the preservation of the superficial sensory nerve?

(ON) Do you systematically prepare the cephalic vein during RFFF harvest?
• Yes
• No

(OP) How does the preparation of the cephalic vein contribute to the success of the procedure in your experience?

(ON) Do you systematically save the fat between the cephalic vein and the pedicle during RFFF harvest? (Yes/No)
• Yes
• No

(OP) What are the considerations guiding your decision on preserving or removing this fat?

(M) How do you monitor the flap's viability during the harvesting process?
• Visual assessment
• Doppler
• ICG angiography
• Other (please specify)

(OP) Are there specific signs or parameters you closely observe to ensure adequate perfusion?

(OP) How do you determine the appropriate length of the pedicle during RFFF harvest?

(OP) At what stage in the procedure do you typically divide the pedicle?

(ON) In cases where the sensory nerve is sacrificed, do you perform nerve grafting during RFFF harvest?
• Yes
• No

(OP) What considerations guide your decision on whether to perform nerve grafting?

(OP) How do you manage potential complications during or after RFFF harvest, such as vascular compromise or nerve injury?

(M) How do you typically close the defect following RFFF harvest?
• Primary closure
• Split-thickness skin graft
• Full-thickness skin graft
• Local flap
• Tissue expander
• Combination of techniques
• No closure (healing by secondary intention)
• Other (please specify)

(OP) Are there specific patient factors that influence your choice of closure technique?

(ON) What type of dressing do you typically employ for forearm wounds?

• Static pressure dressings
• Negative pressure wound dressings

Section IV – Microsurgery

(ON) When performing RFFF, what do you typically use as the primary donor vein for microvascular anastomosis?
• Cephalic vein
• Comitant veins
• Both
• Other (please specify)

(OP) What factors influence your decision to choose the cephalic vein, comitant veins, or both for microvascular anastomosis during RFFF?

(ON) Do you consider the diameter of the cephalic vein or comitant veins when choosing the donor vein for anastomosis?
• Yes
• No

(OP) Are there specific diameter thresholds that influence your decision?

(OP) How do you assess the quality of the cephalic vein or comitant veins before deciding on their use as donor veins for anastomosis?

(ON) In cases where comitant veins are used, do you perform sequential anastomosis of multiple veins during RFFF harvest?
• Yes
• No

(OP) What factors influence your decision to perform sequential anastomosis?

(OP) Are there specific techniques you employ to prepare vessels for microvascular anastomosis during RFFF?

(OP) Do you prefer end-to-end or end-to-side anastomosis? Why?

(OP) What factors influence your choice of anastomotic technique in different cases?

(OP) What type of suture do you prefer?

(OP) What type of suturing technique do you prefer?

(OP) Are there any modifications or adaptations you make based on patient characteristics or specific surgical challenges?

(OP) What type of technical support (e.g., microscope, exoscope, loupes) do you use to perform anastomoses?

(OP) How important is technical support in ensuring successful microsurgical outcomes?

Section V – Postoperative care

(ON) Do you administer antibiotics perioperatively for patients undergoing radial forearm free flap surgery?
• Yes
• No

(OP) If yes, what is the duration of antibiotic administration?

(OP) What indications prompt the use of antibiotics in your postoperative care protocol?

(ON) Do you incorporate anticoagulation in the postoperative management of radial forearm free flap patients?
• Yes
• No

(OP) If yes, what type of anticoagulant drug is typically administered?

(OP) Could you provide detailed information on the dosage of the anticoagulant used?

(OP) When do you initiate anticoagulation after the surgery (e.g., immediate post-op, postoperative day 1)?

(OP) What is the recommended duration of anticoagulant intake in your protocol?

Section VI – Flap monitoring modalities

(M) Who is responsible for postoperative flap monitoring?
• Primay surgeon
• Surgical resident
• Nursing staff
• Dedicated microsurgical nurse
• Anesthesiologist
• Multidisciplinary team (Surgeon, Nurse, Anesthesiologist)
• Other (please specify)

(OP) How frequently do you follow up with patients who have undergone RFFF procedures?

(M) What monitoring strategies do you use to identify potential issues early and prevent complications?
• Clinical assessment (regular clinical examination of the flap, including color, temperature, capillary refill, and overall tissue perfusion)
• Doppler ultrasound
• Continuous Wave Doppler Probe
• Near-infrared spectroscopy (NIRS)
• Intraoperative Indocyanine Green (ICG) Angiography
• Infrared thermometry
• Capnography
• Bioimpedance Spectroscopy
• Tissue pH monitoring devices
• Transcutaneous Oxygen Tension (TcPO2)
• Other (please specify)

(OP) Are there specific patient factors or clinical scenarios that influence your choice of a particular type of monitoring strategy?

Section VII – Outcomes

(OP) How would you rate the overall success of RFFF procedures in terms of flap survival?

(OP) What is your estimated rate of revision surgeries in your practice?

(OP) What factors contribute to the need for revisions in RFFF procedures, in your experience?

(OP) Can you provide an estimate of the overall loss rate for RFFF in your practice?

(OP) What are the primary reasons for the loss of free flaps, as observed in your surgical cases?

(OP) To what extent do you collaborate with other specialties (e.g., vascular surgery, anesthesia) in planning and executing free flap procedures?

(OP) How does a multidisciplinary approach influence the outcomes and complication rates in free flap surgeries?

(OP) In your experience, what is the typical postoperative recovery time for patients who undergo RFFF procedures?

(OP) How do you assess the functional outcomes of RFFF in terms of speech and swallowing?

(OP) How do you evaluate the aesthetic outcomes of RFFF in head and neck reconstruction?

(OP) How do you assess patient satisfaction following RFFF procedures?

(OP) In your experience, do patient-reported outcomes align with clinical assessments of flap success?
